# Long-Term Sequelae of COVID-19 in Experimental Mice

**DOI:** 10.1007/s12035-022-02932-1

**Published:** 2022-07-13

**Authors:** Michael J. Paidas, Daniela S. Cosio, Saad Ali, Norma Sue Kenyon, Arumugam R. Jayakumar

**Affiliations:** 1 Department of Obstetrics, Gynecology and Reproductive Sciences, University of Miami Miller School of Medicine, 1120 NW 14th Street, Suite # 1154, Miami, FL 33136 USA; 2 Department of Pathology and Laboratory Medicine, University of Miami Miller School of Medicine, Miami, FL 33136 USA; 3 Microbiology & Immunology and Biomedical Engineering, Diabetes Research Institute, University of Miami, Miami, FL USA

**Keywords:** COVID-19, Long-term sequelae, mice, Multi-organ histopathology, Mouse hepatitis virus-1, SARS-CoV-2, Vascular defect

## Abstract

We recently reported acute COVID-19 symptoms, clinical status, weight loss, multi-organ pathological changes, and animal death in a murine hepatitis virus-1 (MHV-1) coronavirus mouse model of COVID-19, which were similar to that observed in humans with COVID-19. We further examined long-term (12 months post-infection) sequelae of COVID-19 in these mice. Congested blood vessels, perivascular cavitation, pericellular halos, vacuolation of neuropils, pyknotic nuclei, acute eosinophilic necrosis, necrotic neurons with fragmented nuclei, and vacuolation were observed in the brain cortex 12 months post-MHV-1 infection. These changes were associated with increased reactive astrocytes and microglia, hyperphosphorylated TDP-43 and tau, and a decrease in synaptic protein synaptophysin-1, suggesting the possible long-term impact of SARS-CoV-2 infection on defective neuronal integrity. The lungs showed severe inflammation, bronchiolar airway wall thickening due to fibrotic remodeling, bronchioles with increased numbers of goblet cells in the epithelial lining, and bronchiole walls with increased numbers of inflammatory cells. Hearts showed severe interstitial edema, vascular congestion and dilation, nucleated red blood cells (RBCs), RBCs infiltrating between degenerative myocardial fibers, inflammatory cells and apoptotic bodies and acute myocyte necrosis, hypertrophy, and fibrosis. Long-term changes in the liver and kidney were less severe than those observed in the acute phase. Noteworthy, the treatment of infected mice with a small molecule synthetic peptide which prevents the binding of spike protein to its respective receptors significantly attenuated disease progression, as well as the pathological changes observed post-long-term infection. Collectively, these findings suggest that COVID-19 may result in long-term, irreversible changes predominantly in the brain, lung, and heart.

## Introduction

Infection with SARS-CoV-2 causes a respiratory illness and severely affects other organ systems [1–4], possibly precipitated by cytokine storm, septic shock, thrombosis, and oxidative/nitrative stress [5–7]. There have been more than 460 million cases, over 6.0 million deaths, and a 7-day average of approximately 3 million new cases worldwide as of March 2022. Furthermore, the Food and Drug Administration has only approved remdesivir and two other medications, under emergency use authorization, to treat SARS-CoV-2 infection in all age groups/populations.

We recently reported COVID-19 symptoms, clinical status, weight loss, and animal death in a murine hepatitis virus-1 (MHV-1) coronavirus mouse model of COVID-19 [1, 8–10], which were similar to those observed in humans with SARS-CoV-2 infection.

A wide range of viral entry mechanisms, as well as immunological and pathological changes, have been identified in humans with COVID-19 post-acute infection with SARS-CoV-2 [11–19]. These include an imbalance in angiotensin-converting enzyme-2 (ACE-2) and its regulation and altered immune response and inflammatory processes (innate and adaptive immune response, autoimmunity, severe inflammation, and host-specific factors) [11–19]. While the pathological changes reported in these patients are predominantly in the lung, multi-organ failure has also occurred in almost all cases [1–4]. Changes identified in the lungs of patients with acute SARS-CoV-2 infection demonstrate major diffuse alveolar damage [20–23]. However, it should be highlighted that hospitalized patients who died of acute SARS-CoV-2 infection with high viral load had less severe lung pathology than those who had the infection for a longer period (severe lung damage) suggesting the possible long-term defect in the lung. Similarly, immune, inflammatory, and pathological changes have been identified in other organs including the brain, liver, heart, kidney, and gastrointestinal system [24–28], although the viral entry mechanisms in these organs are not well defined. Major common immune, inflammatory, and pathological changes observed after acute infection with SARS-CoV-2 in these organs include severe inflammation, infiltration of inflammatory cells, congested blood vessels, microvascular thrombosis, and edema [29–32].

While studies on various tissues from people who died of COVID-19 provide evidence of pathological changes in the acute phase, there is limited evidence available on the long-term effects of SARS-CoV-2 infection. Recent reports indicate that patients who survived after acute severe SARS-CoV-2 infection experience breathing difficulties [33], heart problems (inflammation of the heart muscle and increased heart rate, [34], and damage to the kidney [35], suggesting the possible lifelong defects in multiple organs post-SARS-CoV-2 infection. Therefore, we examined the long-term sequelae of COVID-19 using the MHV-1 mouse model of COVID-19, which we have established [1, 8–10]. While our findings show a partial recovery in the liver and kidney long-term after infection, severe pathological changes in the brain, lung, and heart were observed 1-year post-MHV-1 coronavirus infection. These findings suggest possible irreversible multi-organ complications in COVID-19.

## Materials and Methods

Female A/J mice (8 weeks of age, weighing 22 g) were purchased from Jackson Laboratories (Bar Harbor, ME) and were maintained in micro-isolated cages, housed in the animal colony at the Biomedical Research Building animal isolation facility at the University of Miami Miller School of Medicine. They were fed a standard lab chow diet [Envigo 2918 irradiated (Teklad diet, Dublin, VA)] and provided autoclaved tap water ad libitum. The study was conducted according to the guidelines of the University of Miami Institutional Animal Care and Use Committee (IACUC protocol number 20–131 LF) approved on October 8, 2020.

### Viral Inoculation and Experimental Group

MHV-1 was purchased from American Type Culture Collection (ATCC, cat# VR-261, Manassas, VA). Mice were inoculated with 5000 PFU intranasally [1, 8–10]. Briefly, 5 × 10^3 ^PFU MHV-1 was mixed with 50 μl of ice-cold Dulbecco’s Modified Eagle’s Medium (DMEM, Gibco Cat# 11,965–092, Lot# 2,186,816, Thermo Fisher Scientific, Waltham, MA) and instilled into the nares immediately, and mice were observed until the virus was inhaled. The mice were divided into 5 groups (3 mice in each): (1) healthy controls, (2) infusion of healthy controls with DMEM (used for intranasal infusion of MHV-1), (3) infusion of MHV-1 alone, and (4) infusion of MHV-1 + SPIKENET (SPK, a small molecule synthetic peptide which prevents the binding of spike protein to its respective receptors), and (5) SPK alone. SPK (3 doses of 5 mg/kg) was chosen based on our earlier study in acute infection [36] and injected subcutaneously in MHV-1-inoculated mice every alternate day from day 2 (i.e., 2, 4, and 6 days post-MHV-1).

### Clinical Observation

Mice inoculated with MHV-1 were monitored for clinical signs of disease as described earlier [1, 8–10]. Clinical signs were scored by stages of (0) no clinical signs, (I) drowsy and lack of movement; (II) slightly ruffled fur and altered hind limb posture; (III) ruffled fur and mildly labored breathing; (IV) ruffled fur, inactive, moderately labored breathing; (V) ruffled fur, labored breathing, and lethargy; and (VI) moribund and death.

Mice (12 months post-infection) were weighed and euthanized, and their major organs, including brains, lungs, livers, kidneys, and hearts, were removed and fixed in 10% formalin, processed routinely for paraffin sections and stained with hematoxylin and eosin (H&E). Briefly, after gradient dehydration with various concentrations of alcohol in an automatic tissue dehydrator (HistoCore PELORIS 3 Premium Tissue Processing System, Leica Biosystems Inc, Buffalo Grove, IL), tissues were embedded in paraffin blocks by a paraffin embedding station (HistoCore Arcadia Embedding Center, Leica Biosystems Inc, Buffalo Grove, IL). The tissues were then cut into 10-μm-thin slices by an ultra-thin semiautomatic microtome (Histocore autocut automated rotary microtome, Leica Biosystems Inc, Buffalo Grove, IL) and adhered to the slides. After the slides were stained with H&E, morphological changes were evaluated with a microscope (*Olympus VS120* Automated *Slide Scanner, Olympus*, Pittsburgh, Pennsylvania) by a pathologist unaware of the treatment protocol.

To ascertain the extent of liver failure post-long-term virus exposure and potential recovery after the administration of SPK, blood was collected via cardiac puncture, and serum was used to measure liver enzymes. Levels of aspartate aminotransferase (AST), alanine aminotransferase (ALT), alkaline phosphatase (ALP), and bilirubin content were determined 12 months post-infection, as previously described, using a Cobas 0501 automatic analyzer (Roche Diagnostics, IN) [37]. Bodyweight was measured frequently as demonstrated in the results section (see below).

### Examination of Neuronal Proteins/Markers by Immunohistochemistry (Immunofluorescence)

The brain appears to be severely affected, as compared to other organs, in our pathological findings 12 months post-MHV-1 infection (see “Results” section). Additionally, researchers have speculated that COVID-19 accelerates neurological conditions (e.g., Alzheimer’s disease, amyotrophic lateral sclerosis, Parkinson’s disease) and might contribute directly to the development of these conditions [38–43]. Accordingly, we tested whether changes occurred in one or more major neuronal markers that have been strongly implicated in these neurological conditions, in MHV-1-inoculated mice. These include the status of glial cell activation (astrocytes and microglia), hyperphosphorylated TDP-43 and tau, as well as changes in neuronal protein synaptophysin-1. Briefly, paraffin-embedded brain tissue sections from healthy control and MHV-1-inoculated mice (10 microns), with and without SPK treatment, were incubated with anti-TDP-43 Phospho (Ser409/410) Antibody [cat# 829,901, 1:100 dilution, BioLegend (San Diego, CA, USA) (previously Covance catalog# SIG-39852)], Phospho-PHF-tau pSer202 + Thr205 Antibody [(AT8), Life Technologies Corporation **(**cat**#** MN1020, 1:100 dilution)], anti-synaptophysin antibody (rabbit monoclonal, YE269, 1: 150 dilution, cat# 32,127, Abcam, Cambridge, MA, USA), ionized calcium-binding adapter molecule 1 (Iba1) (FL-147: sc-98468, 1:200 dilution, Santa Cruz Biotechnology, Inc. Dallas, TX, USA), and purified mouse Anti-GFAP (Cat# 556,328, 1:100 dilution, BD Pharmingen™, BA1 1BE UK). Sections were washed and incubated in respective Alexa Flour-488/Alexa Flour-546 goat anti-mouse/rabbit or appropriate IgG (H + L) (Life Technologies) and were used at a concentration of 1:200. Immunofluorescent images were acquired with a Zeiss LSM510/UV Axiovert 200 M confocal microscope (Carl Zeiss Microscopy, LLC, Thornwood, NY, USA) with a Plan Apochromat 40 × objective lens and 2 × zoom, resulting in images of 125 × 125 μm in area and 1.0-μm optical slice thickness (1.0 Airy units for Alexa Fluor 546 or 568 emission channel). A random collection of images from sections of control and MHV-1-inoculated mice were obtained by systematically capturing each image in a “blinded” manner by moving the microscope stage approximately 5 mm in four different directions. At least 14 fluorescent images were captured per mouse. Images were quantified using the Volocity 6.0 High-Performance Cellular Imaging Software (PerkinElmer, Waltham, MA, USA) as described previously [44, 45], and normalized to the number of DAPI-positive cells, as well as to the area and intensity of DAPI.

### RNA Isolation and RT-qPCR

Isolation of mRNA was performed as previously described [46, 47]. In brief, the RNAqueous®-4PCR kit (#AM1914; Ambion, Austin, TX, USA) was used for the RNA isolation. High-Capacity cDNA Reverse Transcription Kit (catalog# 4,368,814; Applied Biosystems, Foster City, CA, USA) was used to generate cDNA. The Mx3005P Multiplex Quantitative PCR System (catalog # 401,513; Stratagene/Agilent Technologies, Wilmington, DE, USA), using RT-qPCR SYBR GREEN Reagents (Brilliant® II SYBR® Green QPCR Master Mix; Agilent Technologies) with ROX as the reference dye (final reaction volume 25 μL) was used for RT-qPCR analysis. RT-qPCR cycling conditions included an initial 95 °C for 10 min, 40 cycles of 95 °C for 30 s, 58 °C for 30 s, and 72 °C for 15 s. MxPro-Mx3005P v4.10 software (Stratagene/Agilent Technologies) was used to determine the crossing points for each amplification reaction. NMDAR1 Forward primer 5′-GCAAGAATGAGTCAGCCCAC-3′ and reverse primer 5′-CAGTCACTCCGTCCGCATAC-3′. All RT-qPCR data were normalized against glyceraldehyde 3-phosphate dehydrogenase. Each RT-PCR experiment was repeated at least three times to document its reproducibility. Negative controls included samples in which the cDNA templates were replaced by nuclease-free water in the reactions.

### Statistical Analysis

Data were subjected to analysis of variance followed by Tukey’s multiple comparison test. A statistical analysis showing *p* < 0.05 was considered significant.

## Results

### Bodyweight Measurement, Liver Enzyme Analysis, and Clinical Observation for Long-Term Post-MHV-1 Infection

**Fig. 1:**
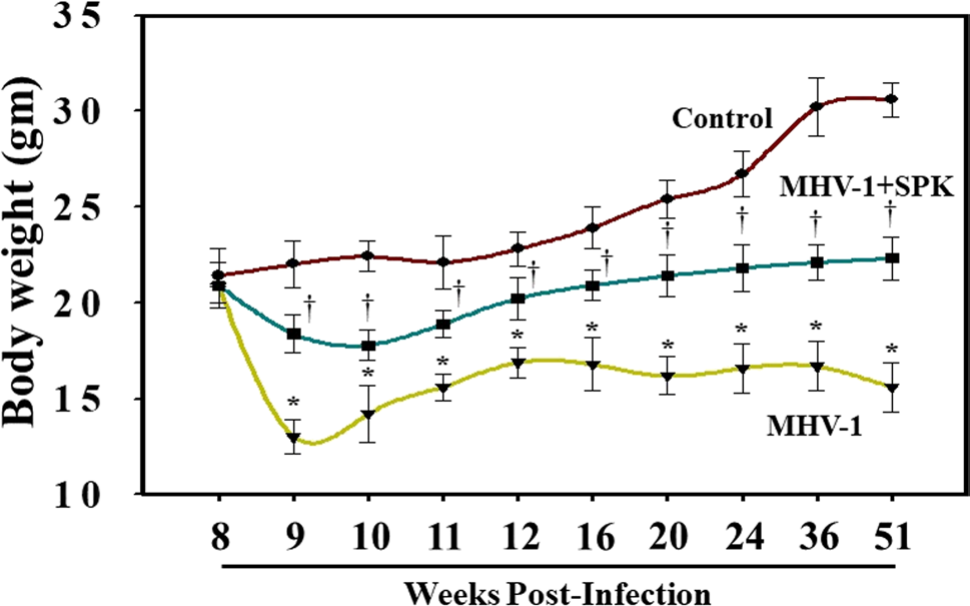
LongtTerm alterations in bodyweight post-MHV-1 infection. MHV-1-infected mice lost body weight in a biphasic manner. Acute loss was identified from 4 to 12 days post-inoculation of 8 weeks old mouse, and such loss was reduced from 3 to 12 weeks (graph 11–24 weeks). Furthermore, a gradual decrease was observed until 11 months and 21 days (51 weeks). Treatment of these mice with SPIKENET (SPK, 5 mg/kg, 3 doses on days 2, 4, and 6 post-MHV-1 inoculation) significantly reduced the weight loss. Data were subjected to ANOVA (*n* = 3). *p < 0.05 vs. control; †*p* < 0.05 vs. MHV-1 alone. Error bars, mean ± S.E

MHV-1-infected mice bodyweight showed a biphasic effect. An initial loss in bodyweight was observed from days 4 to 12. This acute bodyweight loss began to recover at 3 weeks but never rose back to pre-MHV-1-inoculation levels (Fig. 1). A 40% weight gain was observed in MHV-1-inoculated mice, as compared to uninfected mice (i.e., regular weight gain 100%) until 3 months, while there was no additional weight gain until 12 months in these mice (Fig. 1). Treatment of the infected mice with SPK, which prevents the binding of spike protein to its respective receptor (3 doses on days 2, 4, and 6 with 5 mg/kg post-infection) resulted in significant improvement in weight gain, although not to the level that controls animals attained. In this study, we considered the DMEM-inoculated mice as the control for the viral and SPK-treated groups because we did not find any difference between DMEM-inoculated, non-inoculated, and SPK-only groups (figures not shown). The DMEM-inoculated group was also considered a control in the following studies.

**Fig. 2:**
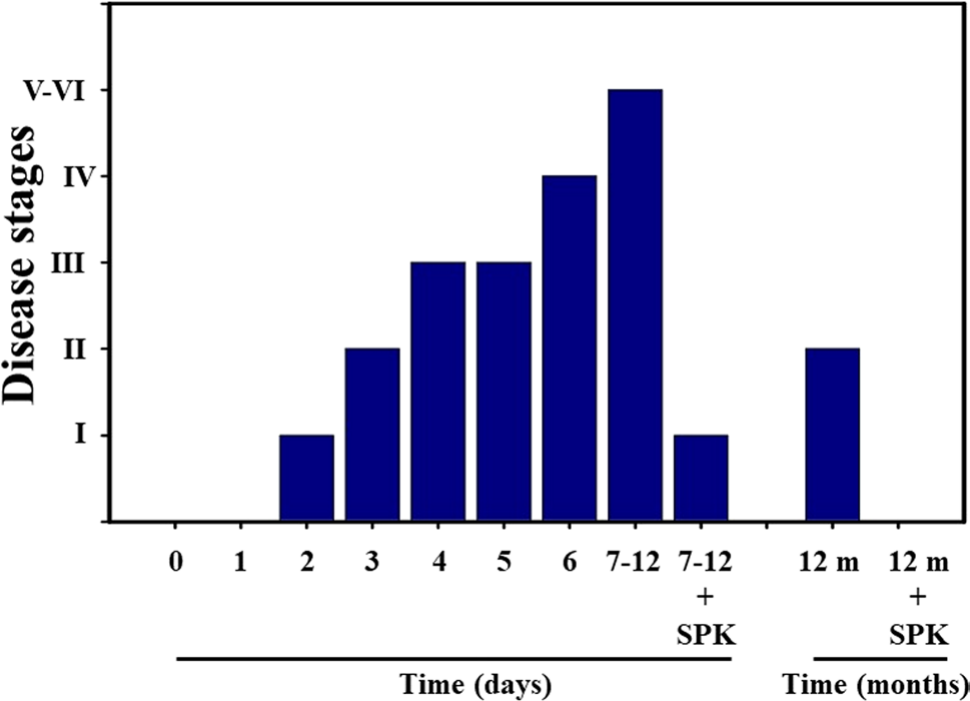
Long-term clinical signs in MHV-1-infected mice. MHV-1-inoculated mice exhibit severe illness in the acute phase (stages IV–VI, from 7 to 12 days), while the animal sickness was significantly reduced to stages II after 12 months. Furthermore, SPIKENET (SPK, 5 mg/kg, 3 doses on days 2, 4, and 6 post-MHV-1 inoculation) prevented such effect in both acute phase and after long-term post-infection

While the levels of liver enzymes were slightly increased after 12 months post-infection, these increases were far less than those observed in acute stages of infection (Table 1). Clinical signs of disease 12 months post-infection were also less than those observed in acute stages of infection (stages II–III, drowsy and lack of movement to ruffled fur and mildly labored breathing 12 months post-infection) (Fig. 2). Furthermore, levels of liver enzymes were normalized, and no obvious clinical symptoms of the disease were noted in infected animals that had been treated with SPK (Fig. 2). While it would be useful to analyze neurobehavioral deficits, we did not conduct such tests (various mazes, available in our facility) because these tests would themselves interfere with the level of severe sickness and would complicate the interpretation of the data. However, this limitation can be resolved in the future by investigating the neurobehavioral deficits with more appropriate tools, as well as in animals after sub-acute viral exposure, where obvious minimal to moderate neurobehavioral deficits can be detected.

**Table 1:** Acute/long-term effect of MHV-1 infection on liver enzymes with/without SPIKENET treatment

	Uninfected mice	MHV-1 infected mice (7 day)	MHV-1 infected Mice (12 month)	MHV-1 infected mice (7 day) + SPK	MHV-1 infected mice (12 month) + SPK
AST (units/l ALT (units/l) ALP (units/l) Bilirubin (mg/l)	96.8 ± 14.2 31.5 ± 11.6 589.1 ± 108.7 0.075 ± 0.02	3459.2 ± 684.1* 3068.5 ± 861.3* 986.3 ± 158.4* 0.86 ± 0.2*	412.8 ± 70.9† 316.4 ± 56.1† 610.3 ± 106.8† 0.27 ± 0.05†	608.7 ± 356.8‡ 1029.1 ± 436.7‡ 643.8 ± 94.1‡ 0.23 ± 0.06‡	136.7 ± 31.6§ 264.9 ± 54.7§ 439.7 ± 84.3§ 0.18 ± 0.035§

Mean values ± SD. *Statistically significant difference from uninfected mice. *AST*, aspartate amino transferase; *ALT*, alanine amino transferase; *ALP*, alkaline phosphatase

**Fig. 3:**
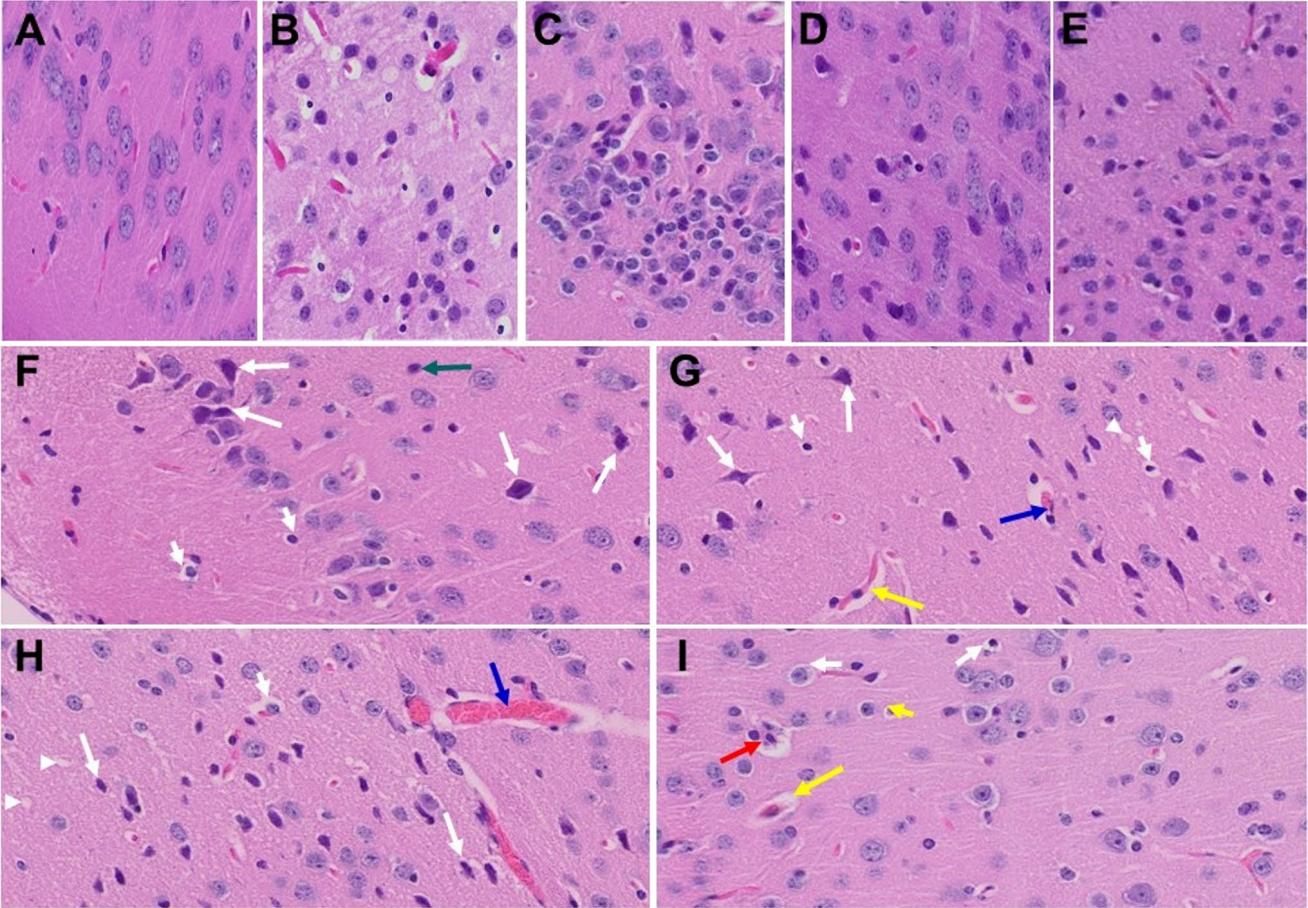
Acute/long-term changes in brain post-MHV-1 coronavirus infection. **A** Normal brain cortex. **B** Representative image from MHV-1-infected mouse brain cortex showed “perivascular cavitation, congested blood vessel, pericellular halos, darkly stained nuclei, vacuolation of neuropil, pyknotic nuclei and acute eosinophilic necrosis at 7 days (acute phase)” [1]. **C** MHV-1-infected mouse brain cortex (12 months post-infection). **F–I** Enlarged images of **C** showed widespread neuronal necrosis (long arrows), pyknotic nuclei/neuronal clearing (short arrows), vacuolation of neuropil (arrowhead), congested blood vessels (blue arrows), perivascular cavitation (yellow arrows, Virchow–robin space), darkly stained nuclei (green arrow), neuronophagia (red arrow, presence of necrotic neurons surrounded by invaded hypertrophic microglia (**I**). **D** and **E** Treatment of MHV-1-infected mice with the peptide drug SPIKENET (5 mg/kg) ameliorated the above-mentioned changes at day 7 and 12 months post-infection, respectively. These findings suggest that the changes observed in the brain post-long-term infection are more severe than in the acute phase and the peptide SPIKENET has therapeutic potential in reducing MHV-1 infection (*n* = 3). (H&E, original magnification 400 × (**A–E**), and **F–I** are enlarged images of **C**)

In our earlier short-term study, upon examination of the MHV-1-infected mice brains, we observed various pathological changes (Fig. 3B, as compared to control A) [1]. Examination of the brains of MHV-1-inoculated mice 12 months after infection showed widespread necrotic neurons with fragmented nuclei and vacuolation in addition to the observation in acute injury (i.e., congested blood vessels, perivascular cavitation, cortical pericellular halos, vacuolation of neuropils, darkly stained/pyknotic nuclei, acute eosinophilic necrosis) [1] (Fig. 3). These findings strongly suggest that the long-term pathological changes observed in the brain post-infection are irreversible, which may cause severe neurodegeneration. Furthermore, treatment of these mice with SPK (5 mg/kg) reduced these changes (Fig. 3).

**Fig. 4:**
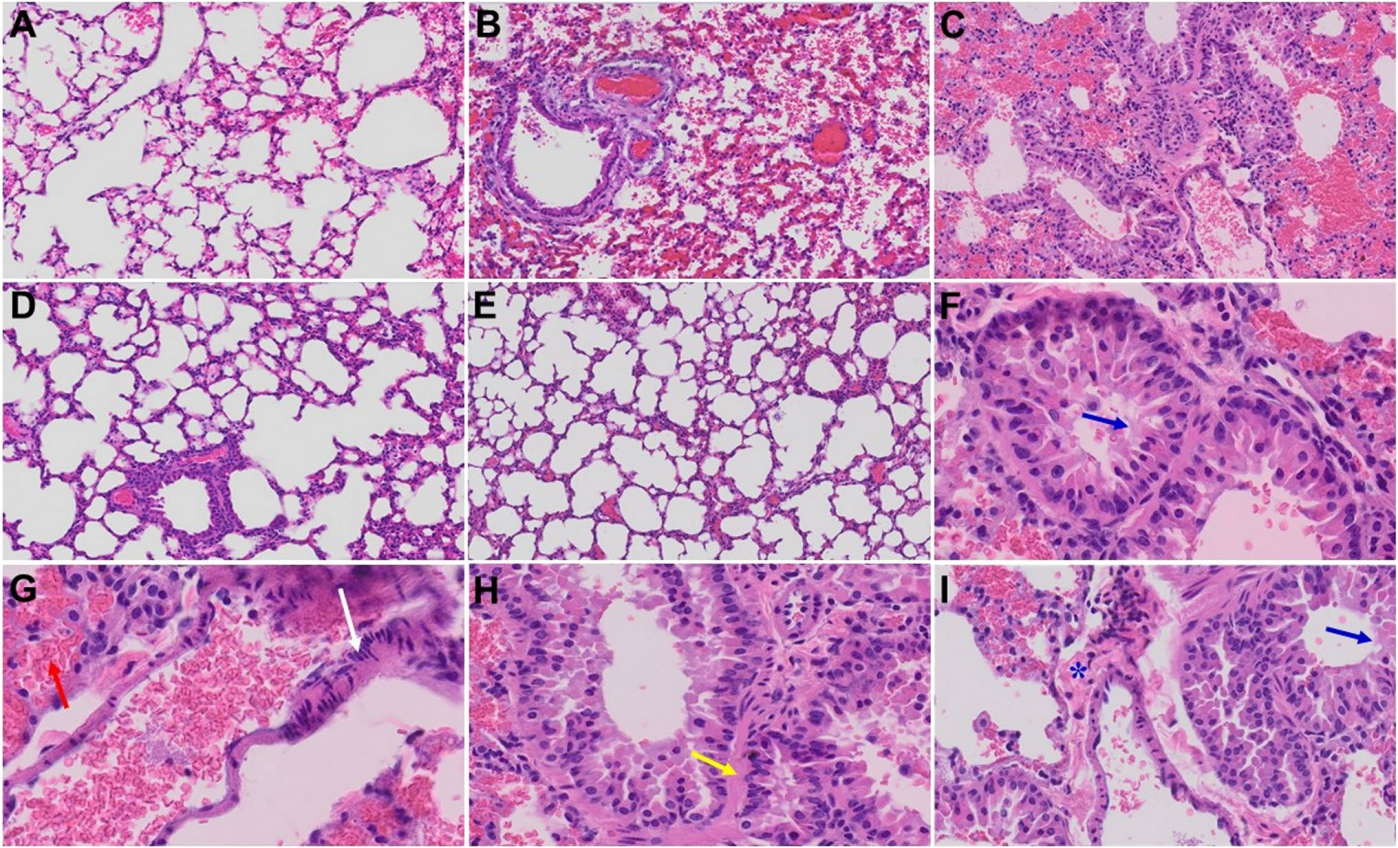
Acute and long-term changes in lung post-MHV-1 coronavirus infection. **A** Normal mouse lung. **B** Representative image from MHV-1-infected mouse lung showed “inflammation (i.e., granular degeneration of cells, and migration of leukocytes into the lungs), along with proteinaceous debris filling of the alveolar spaces with fibrillar to granular eosinophilic protein strands caused by the progressive breakdown of the capillary wall and epithelial integrity, permitting leakage of protein-rich edematous fluid into the alveoli, and the presence of hemosiderin-laden macrophages (indicating pulmonary congestion with dilated capillaries and leakage of blood into alveolar spaces). Furthermore, peribronchiolar interstitial infiltration, bronchiole epithelial cell necrosis, necrotic cell debris within alveolar lumens, alveolar exudation, hyaline membrane formation, alveolar hemorrhage with red blood cells within the alveolar space, and interstitial edema, characteristic features of infected lungs in humans with SARS-CoV-2 infection are observed in MHV-1-infected mice at acute phase (at 7 days [1])”. **C** MHV-1-infected mouse lung (12 months post-infection). **F–I** enlarged images of **C.** Blue arrows, airspaces of alveolar ducts, and alveoli are lined by hyaline membranes; yellow arrow pulmonary edema located in bronchiolar and alveolar airspaces, along with congestion of capillaries in the septal wall, and in the perivascular interstitial spaces; white arrow, nuclear atypia and lack of polarity. Asterisks, intraluminal fibrosis. **D** and **E** Treatment of MHV-1-infected mice with the peptide drug SPIKENET (5 mg/kg) ameliorated the above-mentioned changes at day 7 and 12 months post-infection, respectively (*n* = 3). (H&E, original magnification 400 × (**A–E**), and **F–I** are enlarged images of **C**)

Similarly, in our prior short-term study, we reported that MHV-1-infected mice on day 7 showed severe lung inflammation (Fig. 4B, as compared to control A) [1]. We now found that 12 months after MHV-1 infection, mice still exhibit many of the same acute findings (severe lung inflammation, peribronchiolar interstitial infiltration, bronchiolar epithelial cell necrosis and intra-alveolar necrotic debris, alveolar exudation, mononuclear cell infiltration, hyaline membrane formation, the presence of hemosiderin-laden macrophages, as well as interstitial edema), but also some new findings including bronchioles with thickened airway walls due to fibrotic remodeling (likely due to excessive deposition of collagen bundles), bronchioles containing a large intra-luminal mucous plug, bronchioles with increased numbers of goblet cells in the epithelial lining, and bronchiole walls with increased numbers of inflammatory cells (Fig. 4). Since many of these changes are characteristic features of chronic obstructive pulmonary disease (COPD), it is possible that patients who have been infected with SARS-CoV-2 will experience COPD in the future. Of note, considerable reversal of these changes was observed when these mice were treated with SPK (5 mg/kg) (Fig. 4).

**Fig. 5:**
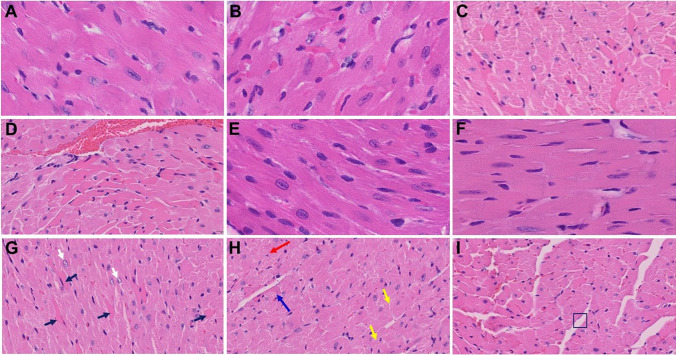
Acute and long-term changes in heart post-MHV-1 coronavirus infection. **A** Heart from normal mice. **B** Representative image from MHV-1-infected mouse heart showed “severe interstitial edema, vascular congestion and dilation, and red blood cells infiltrating between degenerative myocardial fibers after acute infection at 7 days (acute phase)” [1]. **C, D,** and **G–I** MHV-1-infected mouse heart (12 months post-infection). **G** White arrow, enlarged myocytes; block arrows, widespread inflammation. **H** Red arrow, vacuolation; blue arrow, extensive degeneration of cardiac muscle; yellow arrows, foci of apoptotic cell debris; disorganization of the myofibrils with loss of striations. **I** Box showing loss of myocardial fibers. **E** and **F** Treatment of MHV-1-infected mice with the peptide drug SPIKENET (5 mg/kg) ameliorated the above-mentioned changes at day 7 and 12 months, respectively (*n* = 3). (H&E, original magnification 400 × (**A, B, E,** and **F**); and **C, D,** and **G–I** are enlarged images from 400 ×)

We recently reported that the heart of MHV-1-infected mice acutely showed severe interstitial edema, vascular congestion and dilation, and RBCs infiltrating between degenerative myocardial fibers [1] (Fig. 5B, as compared to control A). In our long-term (12-month) examination of MHV-1-infected mice, we found these same outcomes, as well as the presence of inflammatory cells and apoptotic bodies in the cardiac tissue, and acute myocyte necrosis, hypertrophy, and fibrosis (Fig. 5). Such changes were attenuated by treatment of these mice with 5 mg/kg SPK (Fig. 5).

**Fig. 6:**
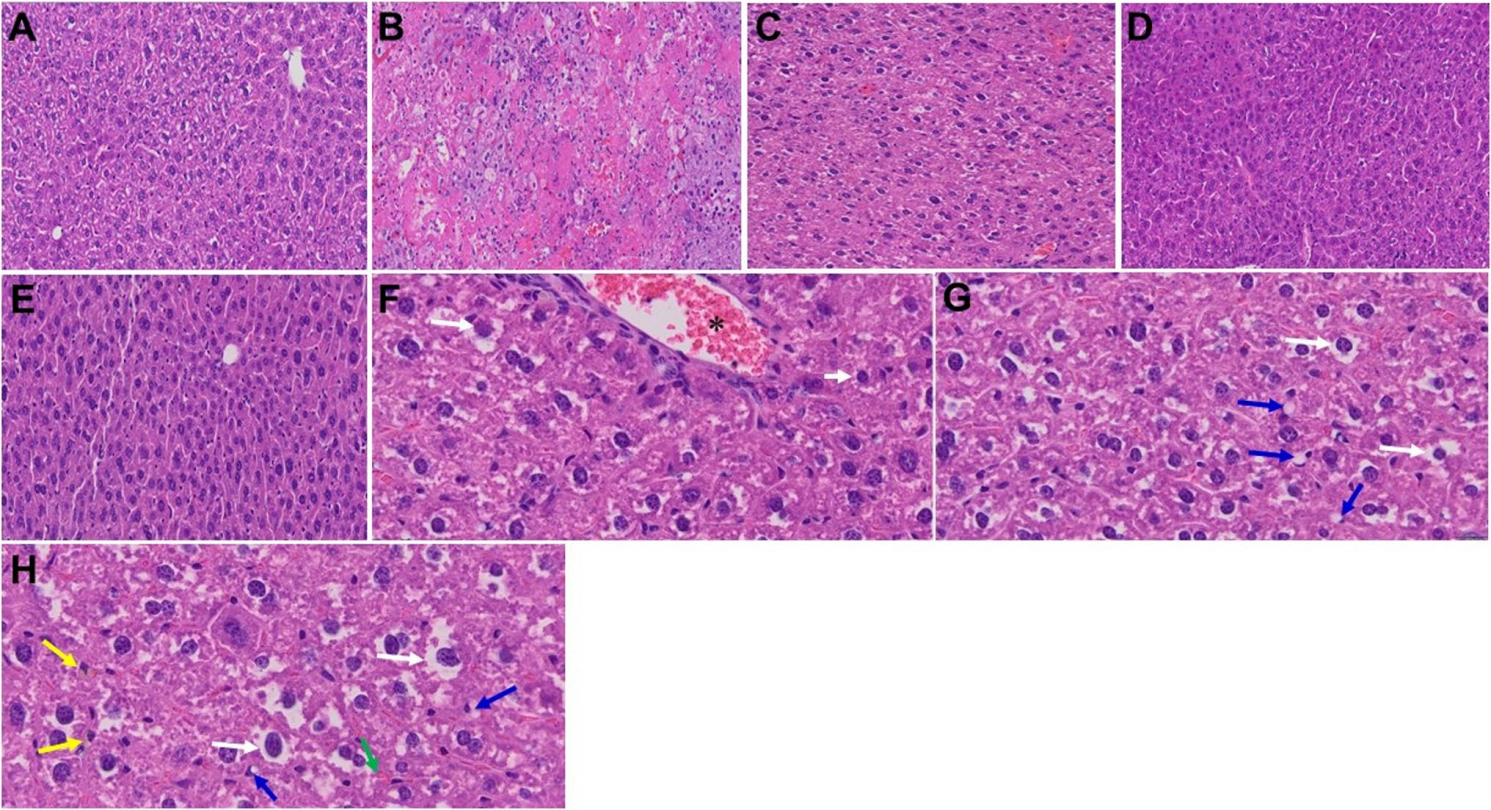
Acute and long-term changes in heart post-MHV-1 coronavirus infection. **A** Liver from normal mice. **B** Representative image from MHV-1-infected mouse liver showed “hepatocyte degeneration, severe periportal hepatocellular necrosis with pyknotic nuclei, severe hepatic congestion, ballooned hepatocytes, vacuolation, and the presence of piecemeal necrosis, as well as hemorrhagic changes. Ground glass hepatocytes showed voluminous, abundant, granular cytoplasm, peripheral cytoplasmic clearing, and central nuclei, and apoptotic (acidophil) bodies, as well as absent hepatocytes replaced by abundant inflammatory cells. Condensation and dark staining of the cytoplasm, an absence of the nucleus, fatty changes, binucleated hepatocytes, and activated Kupffer cells were also identified at 7 days post-infection” [1]. **C** MHV-1-infected mouse liver (12 months post-infection). **F–H** Enlarged images of **C**. Note, the pale brown (yellow arrows in **H**) is lipofuscin pigment (indicative of oxidative stress) that has accumulated as the atrophic and dying cells likely due to hypoxia, undergo autophagocytosis; accumulation of small fat droplets in hepatocyte cytosol (short arrow in **F**); hepatic cells with ballooning degeneration (long arrows in **F–H**); dilated congested blood vessel (asterisk in **F**); presence of stellate cell (Ito cell or perisinusoidal lipocytes) lipidosis (blue arrows) and occasional fat-laden stellate cells showing multiple lipid vacuoles with indentation of the nucleus were also observed (green arrow in **H**). **D** and **E** Treatment of MHV-1-infected mice with the peptide drug SPIKENET (5 mg/kg) ameliorated the above-mentioned changes at day 7 and 12 months post-infection, respectively (*n* = 3). (H&E, original magnification 400 × (**A–E)**, and **F–H** are enlarged images of **C**)

**Fig. 7:**
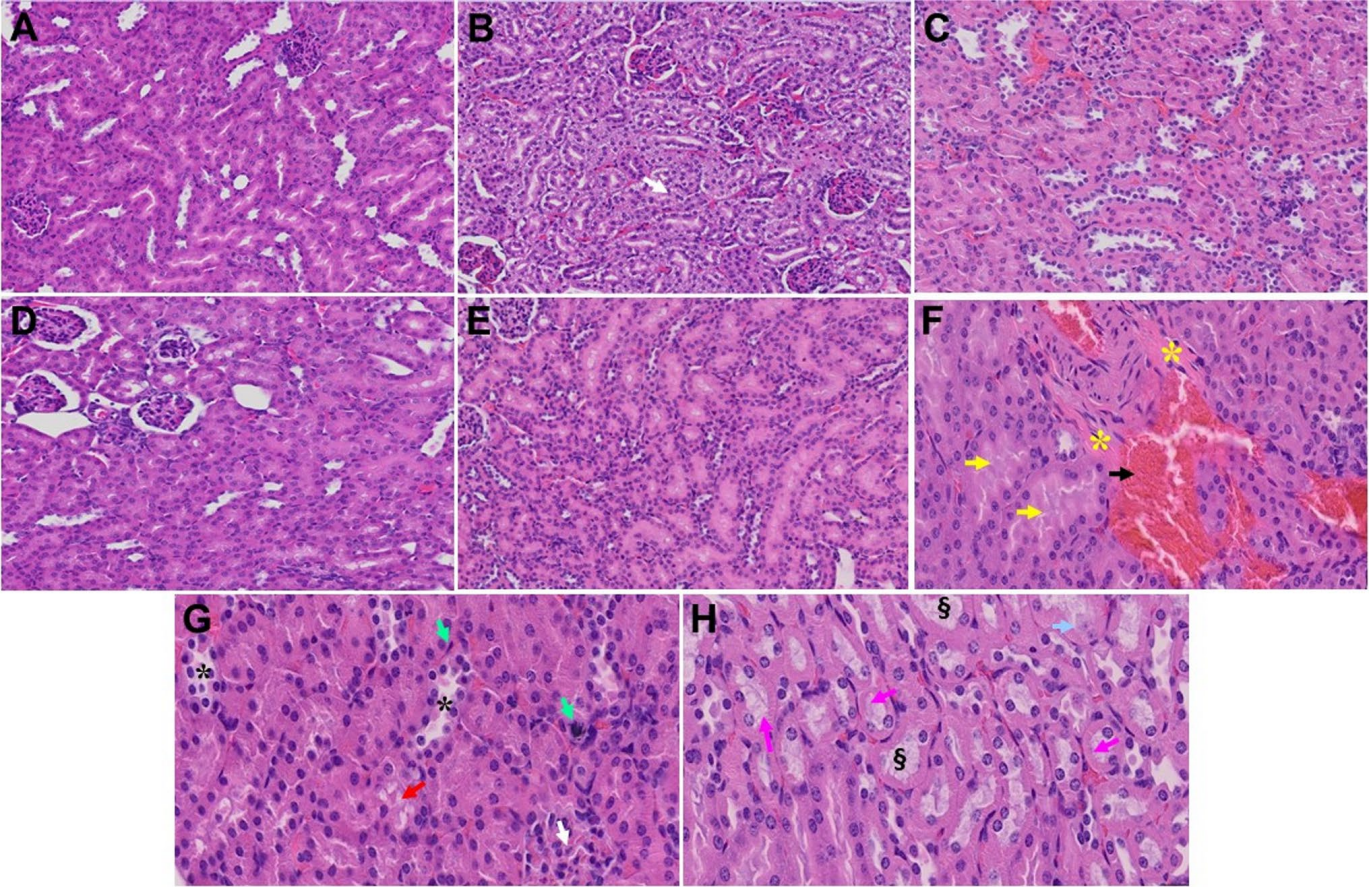
Acute and long-term changes in kidney post-MHV-1 coronavirus infection. **A** Normal mouse kidney. **B** Representative image from “MHV-1-infected mouse kidney showed tubular epithelial cell degenerative changes, peritubular vessel congestion, proximal and distal tubular necrosis, hemorrhage in interstitial tissue, and vacuolation of renal tubules were observed in MHV-1 exposed mice kidneys at 7 days post-infection” [1]. (V). **C** MHV-1-infected mouse kidney (12 months post-infection). **F–H**, enlarged images of **C**, shows congested blood vessels (black arrow), distal tubular damage (yellow arrows), fibrosis and inflamed glomeruli (asterisks), necrosis (green arrows), loss of podocytes (white arrow), degenerating tubules (black asterisks), hyaline casts (pink arrows), loss of tubular epithelial cells (karyolysis) (dagger symbol) and Karyorrhexis (light blue arrow). **D** and **E** Treatment of MHV-1-infected mice with the peptide drug SPIKENET (5 mg/kg) ameliorated the above-mentioned changes at day 7 and 12 months post-infection, respectively (*n* = 3). (H&E, original magnification 400 × (**A-E**), and **F–H** are enlarged images of **C**)

In our prior study, liver from MHV-1-exposed mice (7 days post-infection) showed various pathological changes [1] (Fig. 6B, as compared to control A). In our long-term (12-month) examination of MHV-1-infected mice, we additionally observed increased lymphocyte infiltration in sinusoidal spaces, multifocal hepatic necrosis both in the periportal area and near the terminal hepatic veins, an increased number of portal veins associated with luminal severe dilatation, activated Kupffer cells with large cytoplasm containing necrotic debris, eosinophilic bodies, mitotic cells, and balloon-like liver cells, mild inflammation of lobular lymphocytic and portal tract, and mild hydropic degeneration of liver parenchymal cells (Fig. 6). Treatment of these mice with SPK (5 mg/kg) reduced these changes (Fig. 6). Similarly, in our prior short-term study, we observed various pathological changes in kidneys post-MHV-1 [1] (Fig. 7B, as compared to control A). In addition to the persistence of these acute findings, 12 months after infection, we also identified edema and inflammation of the renal parenchyma, severe acute tubular necrosis, as well as infiltration of macrophages and lymphocytes (Fig. 7). Treatment of these mice with SPK (5 mg/kg) reduced these changes (Fig. 7). These findings collectively suggest that the structural changes in liver and kidney post-SARS-CoV-2 infection may also be irreversible, although some changes in liver and kidney in these mice are less severe than those observed in the acute phase.

**Fig. 8:**
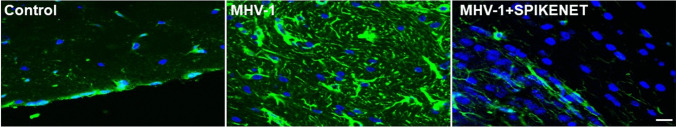
Increased cortical astrogliosis post-MHV-1 coronavirus infection. Representative image from MHV-1 infected mouse (12 months) brain cortex showed severe reactive astrocytes (astrogliosis, green, glial fibrillary acidic protein, GFAP, in MHV-1), as compared to normal mice brain cortex (control). Note, such an increase in astrogliosis was reduced when these mice were treated with the peptide drug SPIKENET (5 mg/kg) (MHV-1 + SPIKENET) (*n* = 3). Scale bar = 35 µm. Blue, nuclear stain DAPI

**Fig. 9:**
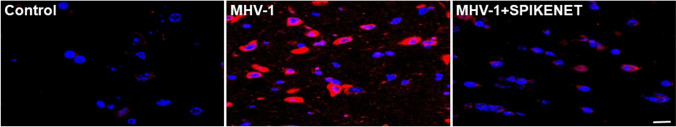
Increased cortical reactive microglia post-MHV-1 coronavirus infection. Representative image from MHV-1 infected mouse (12 months) brain cortex showed severe reactive microglia (red, ionized calcium-binding adaptor molecule 1, Iba1, in MHV-1), as compared to normal mice brain cortex (control). Note, such an increase in reactive microglia was reduced when these mice were treated with the peptide drug SPIKENET (5 mg/kg) (MHV-1 + SPIKENET) (*n* = 3). Scale bar = 35 µm. Blue, nuclear stain DAPI

**Fig. 10:**
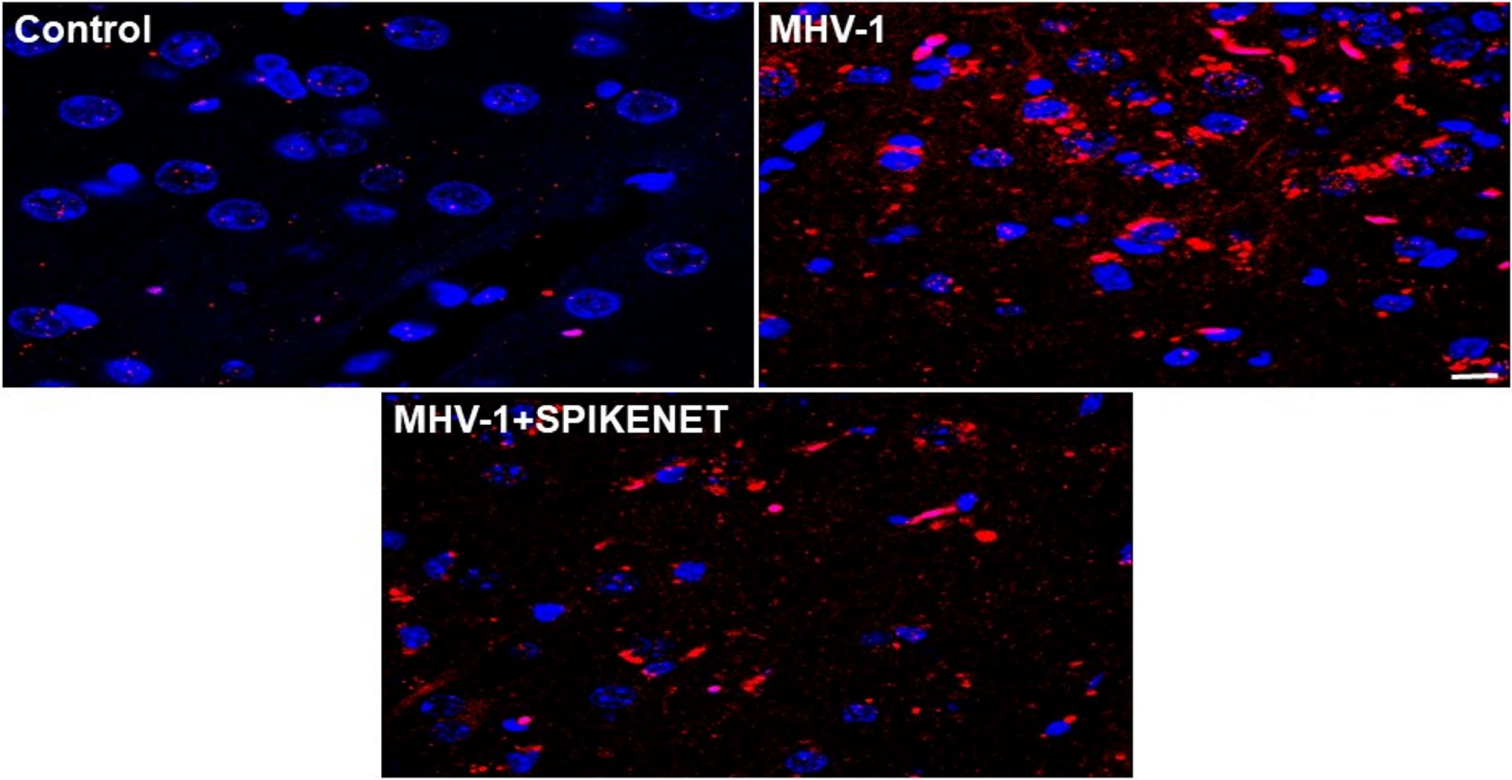
Phosphorylated TDP-43 (p-TDP-43) level in the cerebral cortex of MHV-1-infected mice. Normal (control) brain illustrates the basal level of p-TDP-43 in the brain cortex. Note the marked increase in levels of p-TDP-43 in MHV-1-infected brains (MHV-1) and such increase in p-TDP-43 was reduced when these mice were treated with the peptide drug SPIKENET (5 mg/kg) (MHV-1 + SPIKENET) (*n* = 3). Scale bar = 35 µm. Blue, nuclear stain DAPI

**Fig. 11:**
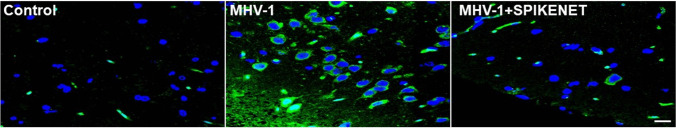
Phosphorylated Tau (p-Tau) level in the cerebral cortex of MHV-1-infected mice. Normal (control) brain illustrates the basal level of p-Tau in the brain cortex. Note the marked increase in levels of p-Tau in MHV-1-infected brains (MHV-1) and such increase in p-Tau was reduced when these mice were treated with the peptide drug SPIKENET (5 mg/kg) (MHV-1 + SPIKENET) (*n* = 3). Scale bar = 35 µm. Blue, nuclear stain DAPI

**Fig. 12:**
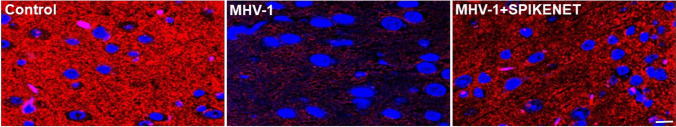
Synaptophysin-1 (Syn-1) level in the cerebral cortex of MHV-1-infected mice. Normal (control) brain illustrates the basal level of Syn-1 in the brain cortex. Note the marked decrease in levels of Syn-1 in MHV-1-infected brains (MHV-1) and such decrease in Syn-1 was reduced when these mice were treated with the peptide drug SPIKENET (5 mg/kg) (MHV-1 + SPIKENET) (*n* = 3). Scale bar = 35 µm. Blue, nuclear stain DAPI

Since long-term pathological changes in the brain post-infection are more severe and the changes are similar to those observed in various chronic neurological conditions, we further examined major neuronal markers including hyperphosphorylated TDP-43 and tau, NR1 subunit of NMDA receptor, astrocytic and microglial activation, and presynaptic protein synaptophysin-1. We found increased reactive astrocytes (Fig. 8), as well as microglia (Fig. 9), in the cerebral cortex of MHV-1-infected mice, and treatment of these mice with 5 mg/kg SPK reduced these changes (Figs. 8 and 9). Similarly, an increase in phosphorylated TDP-43, as well as tau, was observed in these mice, and treatment of these mice with SPK (5 mg/kg) reduced these changes (Figs. 10 and 11, respectively). There was almost a complete loss of synaptophysin-1 observed in the MHV-1-infected mice (Fig. 12), and such loss was diminished by treatment of these mice with SPK (5 mg/kg). These findings strongly suggest the possible long-term sequelae of the brain in COVID-19.

**Fig. 13:**
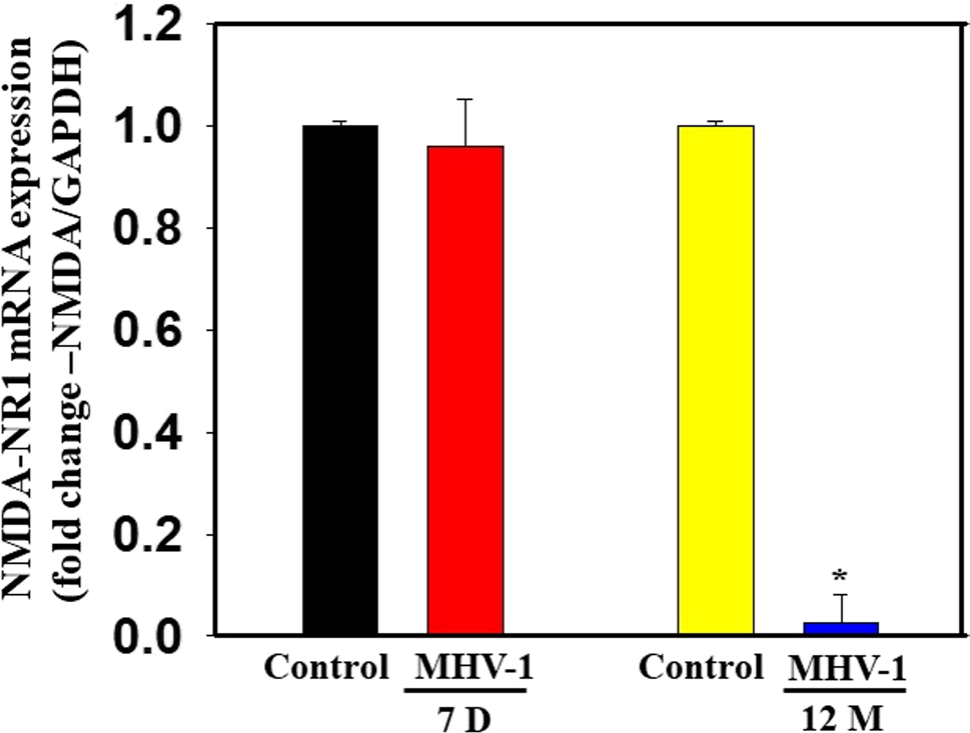
NMDA mRNA in MHV-1-inoculated mice brain cortex. While there was a slight decrease in NR1 subunit of NMDA receptor mRNA at 7 days post-MHV-1, there was a significant loss at 12 months post-MHV-1 infection as determined by RT-qPCR. NMDA mRNA was normalized against glyceraldehyde 3-phosphate dehydrogenase (GAPDH). Data were subjected to ANOVA (*n* = 3). *p < 0.05 vs. control. Error bars, mean ± S.E. D, days; M, months

We also measured NMDA mRNA in MHV-1-inoculated mice brain cortex. While there was a slight decrease in NR1 subunit of NMDA receptor mRNA at 7 days post-MHV-1 (4%), there was a significant loss at 12 months post-MHV-1 infection (98%) as determined by RT-qPCR (Fig. 13). NMDA mRNA was normalized against glyceraldehyde 3-phosphate dehydrogenase (GAPDH).

## Discussion

Our findings demonstrate that MHV-1-infected mice exhibit major long-term pathological changes in various organs, but more so in the brain, lungs, and heart than in the liver and kidney. While some of these long-term changes were new, many were changes that had been observed in the acute phase of infection [1] and seem to have persisted. Furthermore, we now show that the long-term changes in mice brains are associated with increased reactive astrocytes and microglia, hyperphosphorylated TDP-43 and tau, decreased NR1 subunit of NMDA receptor expression, and a decrease in synaptic protein synaptophysin-1 level, suggestive of the possible long-term impact of SARS-CoV-2 infection on defective neuronal integrity. Additionally, treatment of infected mice with a small molecule synthetic peptide, SPK, which prevents the binding of spike protein to its respective receptors, significantly attenuated disease progression. These findings strongly suggest that COVID-19 may result in long-term, irreversible changes, predominantly in the brain, lung, and heart, and that SPK can be considered a novel and effective treatment to prevent COVID-19 (Table 2).

**Table 2:** Differences in acute and long-term changes post-MHV-1 coronavirus infection in mice (highlighted items in 12 months post-infection are important changes that were identified in addition to those observed in mice 7 days post-infection)

	Acute changes (7 days post-infection)	Long-term changes (12 months post-infection)	Post-treatment with SPK (both acute and after long-term)
Clinical signs	Stages IV–VI	Stages II–III	No signs of symptoms
Liver enzymes	AST and ALT, 30–90-fold increase; ALP and bilirubin, 1–tenfold increase	AST and ALT, 4–tenfold increase; ALP and bilirubin, 1–fourfold increase	7 days: AST and ALT, 6–30-fold increase; ALP and bilirubin, 1–threefold increase 12 months: AST and ALT, 1–eightfold increase; ALP and bilirubin, 1–twofold increase
Brain	Congested blood vessels Perivascular cavitation Pericellular halos Vacuolation of neuropils Darkly stained nuclei and pyknotic nuclei amid associated vacuolation of the neuropil Acute eosinophilic necrosis	Congested blood vessels Perivascular cavitation Pericellular halos Vacuolation of neuropils Darkly stained nuclei and pyknotic nuclei amid associated vacuolation of the neuropil Acute eosinophilic necrosis A widespread necrotic neurons with fragmented nuclei and vacuolation	7 days: darkly stained nuclei, occasional perivascular cavitation 12 months: infrequent darkly stained nuclei, moderate pericellular halos, as well as perivascular cavitation
Heart	Severe interstitial edema Vascular congestion and dilation Red blood cells infiltrating between degenerative myocardial fibers	Severe interstitial edema Vascular congestion and dilation Red blood cells infiltrating between degenerative myocardial fibers Presence of inflammatory cells and apoptotic bodies in the cardiac tissue Acute myocyte necrosis Hypertrophy Fibrosis	7 day: mild vascular congestion 12 months: mild vascular congestion
Kidney	Tubular epithelial cell degenerative changes Peritubular vessel congestion Proximal and distal tubular necrosis Hemorrhage in interstitial tissue, and vacuolation of renal tubules	Tubular epithelial cell degenerative changes Peritubular vessel congestion Proximal and distal tubular necrosis Hemorrhage in interstitial tissue, and vacuolation of renal tubules Edema and inflammation of the renal parenchyma Severe acute tubular necrosis Infiltration of macrophages and lymphocytes	7 days: mild-moderate peritubular vessel congestion 12 months: mild peritubular vessel congestion and moderate degenerating tubules
Liver	Hepatocyte degeneration Severe periportal hepatocellular necrosis with pyknotic nuclei Hepatic congestion Ballooned hepatocytes Vacuolation and the presence of piecemeal necrosis Ground glass hepatocytes showed voluminous, abundant, granular cytoplasm, Peripheral cytoplasmic clearing and central nuclei Apoptotic (acidophil) bodies Absent hepatocytes replaced by abundant inflammatory cells Condensation and dark staining of the cytoplasm Absence of the nucleus Fatty changes Binucleated hepatocytes Activated Kupffer cells	Hepatic congestion Ballooned hepatocytes Vacuolation and the presence of piecemeal necrosis Occasional ground glass hepatocytes showed voluminous, abundant, granular cytoplasm, Peripheral cytoplasmic clearing and central nuclei Activated Kupffer cells Lymphocyte infiltration in sinusoidal spaces Multifocal hepatic necrosis both in the periportal area and near the terminal hepatic veins Increased number of portal veins associated with luminal severe dilatation Activated Kupffer cells with large cytoplasm containing necrotic debris Eosinophilic bodies Mitotic cells, and balloon-like liver cells Mild inflammation of lobular lymphocytic and portal tract, Mild hydropic degeneration of liver parenchymal cells lipofuscin pigment (indicative of oxidative stress)	7 days: normal appearance 12 months: mild hepatocyte degeneration
Lung	Inflammation (i.e., granular degeneration of cells, and migration of leukocytes into the lungs) Proteinaceous debris filling of the alveolar spaces with fibrillar to granular eosinophilic protein strands Presence of hemosiderin-laden macrophages Peribronchiolar interstitial infiltration Bronchiole epithelial cell necrosis Necrotic cell debris within alveolar lumens Alveolar exudation Hyaline membrane formation Alveolar hemorrhage with red blood cells within the alveolar space Interstitial edema	Inflammation (i.e., granular degeneration of cells, and migration of leukocytes into the lungs) Proteinaceous debris filling of the alveolar spaces with fibrillar to granular eosinophilic protein strands Presence of hemosiderin-laden macrophages Peribronchiolar interstitial infiltration Bronchiole epithelial cell necrosis Necrotic cell debris within alveolar lumens Alveolar exudation Hyaline membrane formation Alveolar hemorrhage with red blood cells within the alveolar space Interstitial edema Bronchioles with thickened airway walls due to fibrotic remodeling (likely due to excessive deposition of collagen bundles) Bronchioles containing a large intra-luminal mucous plug Bronchioles with increased numbers of goblet cells in the epithelial lining Bronchiole walls with increased numbers of inflammatory cells	7 days: Moderate congested blood vessels 12 months: Mild congested blood vessels

In our earlier acute infection study, upon examination of MHV-1 infected mice brains, we observed: “congested blood vessels, perivascular cavitation (suggestive of edema), pericellular halos, vacuolation of neuropils, darkly stained nuclei and pyknotic nuclei amid associated vacuolation of the neuropil, and acute eosinophilic necrosis” [1]. Examination of the brains of MHV-1-inoculated mice 12 months post-infection showed persistence of almost all of the changes observed in the acute stages of infection, in addition to a wide range of necrotic neurons, strongly suggestive of possible irreversible neurological complications of COVID-19.

In addition to these pathological changes, we also observed increased reactive astrocytes and microglia, hyperphosphorylated TDP-43 and tau, decreased NR1 subunit of NMDA receptor expression, and a decrease in synaptic protein synaptophysin-1 level. While viral infection may directly impact CNS cells that affect these factors, possibly by stimulating hypoxic/ischemic and hemorrhagic lesions [48], these findings may not be specific to direct viral infection on the brain since these effects could be secondary to systemic inflammation and coagulopathy caused by viral infection. In support of the possible direct effect of the virus on the brain, a few studies have shown (1) the presence of SARS-CoV-2 mRNA in cerebrospinal fluid of patients with acute COVID-19 [49, 50], (2) the presence of ACE2 receptors in the blood–brain barrier (BBB) [51, 52], and (3) altered BBB integrity and reduced blood flow, as well as the presence of SARS-CoV-2 in cortical neurons of patients who died of COVID-19 and immune cell infiltrates [48, 53–55]. Since these findings have only been reported in acute stages following SARS-CoV-2 infection and given that these changes may occur either directly or indirectly, it is still unclear how viral infection impacts the brain over a longer period. Our earlier report on an in vitro model of chronic neurological conditions (i.e., chronic traumatic encephalopathy, chronic hepatic encephalopathy) identified similar results to those observed in the long-term study of MHV-1 infected mice brains (e.g., increased TDP-43 and tau phosphorylation) and such changes were found to be mediated by increased levels of casein kinase 1 epsilon (CK1ε), a decrease in importin-β (factors known to mediate the “TDP-43 proteinopathy”) or ubiquitinated and aggregated p-TDP-43 and tau [56, 57]. Furthermore, inhibition of changes in these factors prevented neuronal death [45, 56, 57].

As noted above, brain injury appears to be a major long-term consequence of COVID-19 since we observed a reduction in the level of an important integral membrane glycoprotein, synaptophysin 1, also known as the major synaptic vesicle protein p38, which indicates a loss of synaptic integrity. Such a loss in synaptophysin 1 level may be mediated by hyperphosphorylated TDP-43 and tau, since hyperphosphorylated, and subsequently aggregated, forms of TDP-43 and tau have been strongly implicated in the development of neurodegeneration by altering the level of various neurotransmitters and critical neuronal proteins [57–61]. Additionally, it is possible that astrocytosis and reactive microglia in brains 12 months post-infection stimulate/release inflammatory molecules [62–64] that may increase BBB permeability and recruitment of peripheral immune cells into the brain post-infection for a longer period. It is, therefore, possible that these above-mentioned factors may have been altered in the brain in SARS-CoV-2 infection and that may contribute to long-term neurodegeneration in COVID-19.

Gross section of the lung from SARS-CoV-2-infected patients revealed pulmonary edema and diffusely firm and rubbery parenchyma with no palpable mass [65, 66]. The bronchi were filled with fluid, and there was a predominance of pulmonary congestion and early-stage diffuse alveolar damage with marked hyaline membrane formation, proteinaceous exudates, alveolar hemorrhage, and intra-alveolar fibrin deposition [1]. In addition, there was also a patchy distribution of intra-alveolar foamy macrophages filling some airspace. We also found a similar pattern of pathological changes 7 days post-MHV-1 infection [1]. Furthermore, animals left untreated post-infection for 1 year showed these changes in addition to bronchiolar airway wall thickening caused by fibrotic remodeling (likely due to excessive deposition of collagen bundles), bronchioles containing large intra-luminal mucous plugs, increased numbers of goblet cells in the epithelial lining, and increased numbers of inflammatory cells in the bronchiole walls. These findings strongly suggest that (1) the changes that appeared acutely after infection may not be reversible and (2) the infected animals develop pathological features similar to that of chronic pulmonary disease, suggesting the possible development of chronic pulmonary disease in the future if untreated. Treatment of these mice with our newly created peptide SPK significantly attenuated these deleterious changes.

While acute MHV-1-infected mice hearts showed several pathological changes similar to those observed in humans with COVID-19 in acute stages, such as severe interstitial edema, vascular congestion and dilation, and red blood cell infiltration between degenerative myocardial fibers [1], when left untreated, MHV-1-infected mice hearts, after a year, additionally showed the presence of inflammatory cells and apoptotic bodies in the cardiac tissue and acute myocyte necrosis, myocyte hypertrophy, and fibrosis, which usually appears at the end stages of severe heart disease. Our findings, therefore, demonstrate the lasting cardiac effects of SARS-CoV-2 infection. Noteworthy, both the acute and long-term changes observed in hearts were attenuated by treatment of these mice with SPK (5 mg/kg), suggesting a potential therapy for the treatment of COVID-19.

Livers from SARS-CoV-2-infected patients showed fibrosis, steatosis, congestion, and ischemia, and the most frequently encountered findings were macrovesicular steatosis, mild acute hepatitis, and minimal-to-mild portal inflammation. Interestingly, we found similar changes 7 days post-MHV-1infection in mice [1]. When these mice were left untreated for 1 year, the liver enzyme levels began to normalize; however, one acute phase of pathological change persisted and new pathological changes developed, including increased small lymphocyte infiltration in sinusoidal spaces; multifocal hepatic necrosis both in the periportal area and near the terminal hepatic veins; increased number of portal veins associated with luminal severe dilatation; activated Kupffer cells with large cytoplasm containing necrotic debris, eosinophilic bodies, mitotic cells, and balloon-like liver cells; mild inflammation of lobular lymphocytic and portal tract; and a mild hydropic degeneration of liver parenchymal cells. These findings strongly suggest that the liver is not fully recovered a year after infection despite liver enzyme levels beginning to normalize. Notably, treatment of these mice with SPK (5 mg/kg) reduced these changes, providing a therapeutic option to prevent disease progression.

Pathologic abnormalities of kidneys from postmortem examination of patients with COVID-19 showed loss of proximal tubule brush border, vacuolar degeneration with debris composed of necrotic epithelium in tubular lumens, infiltration of inflammatory cells in tubules and arcuate artery, occasional hemosiderin granules and deposits of calcium in tubules with occasional pigmented casts, and segmental fibrin thrombi present in glomeruli with ischemic glomerular contraction and the accumulation of leaked plasma in Bowman’s space. We found all of these changes acutely in mice that were inoculated with MHV-1 coronavirus [1]. Observation of MHV-1-inoculated mice for a year-long period showed edema and inflammation of the renal parenchyma, severe acute tubular necrosis, and infiltration of macrophages and lymphocytes in addition to the changes observed in the acute phase (7 weeks post-infection). Treatment of these mice with SPK (5 mg/kg) reduced these changes.

Given the severity and permanence of multi-organ involvement in this long-term study of SARS-CoV-2 infection and the similarities observed in the acute effects between MHV-1-inoculated mice and humans with COVID-19, we recommend larger preclinical studies detailing long-term effects of COVID-19 along with human studies when possible. Specifically, examining the role of SARS-CoV-2 infection on long-term brain endothelial cell function and whether SARS-CoV-2 infection results in defective neuronal integrity and long-term neurobehavioral, motor, and cognitive deficits are most important. Detailed evaluation of the lung is also warranted since the lung similarly seems to be severely affected compared to other organs. Additionally, further studies on the peptide used to ameliorate the pathologic changes in this study, along with its pleiotropic effects, are warranted. The multigenerational public health implications of the long-term effects of SARS-CoV-2 infection are enormous, and comprehensive preclinical and clinical strategies are urgently needed.

## Data Availability

The data presented in this study are available on request from the corresponding author. The data are not publicly available due to the University of Miami Miller School of Medicine’s privacy policy.

## Abbreviations

ALP: Alkaline phosphatase
ALT: Alanine aminotransferase
AST: Aspartate aminotransferase
COVID-19: Coronavirus disease 2019
DAPI: 4′,6-Diamidino-2-phenylindole
DMEM: Dulbecco’s Modified Eagle’s Medium
MHV-1: Murine hepatitis virus-1
NMDA: N-methyl-D-aspartate
SPK: SPIKENET (a 15–amino acid synthetic peptide)
SARS-CoV-2: Severe acute respiratory syndrome coronavirus 2
TDP-43: Transactivating DNA-binding protein-43
Tau: Tubulin-binding protein
